# Assessment of Diet and Physical Activity in Paediatric Non-Alcoholic Fatty Liver Disease Patients: A United Kingdom Case Control Study

**DOI:** 10.3390/nu7125494

**Published:** 2015-11-26

**Authors:** Philippa S. Gibson, Sarah Lang, Marianne Gilbert, Deepa Kamat, Sanjay Bansal, Martha E. Ford-Adams, Ashish P. Desai, Anil Dhawan, Emer Fitzpatrick, J. Bernadette Moore, Kathryn H. Hart

**Affiliations:** 1 Nutritional Sciences, Faculty of Health and Medical Sciences, University of Surrey, Guildford GU2 7XH, UK; pippa.s.gibson@gmail.com (P.S.G.); lang.sarah16@gmail.com (S.L.); marianne-gilbert@hotmail.com (M.G.); 2 Department of Nutrition and Dietetics, Monash University, Melbourne, 3168, Australia; 3 Paediatric Liver Centre, King’s College London School of Medicine at King’s College Hospital, London SE5 9RS, UK; deepa.kamat@nhs.net (D.K.); sanjay.bansal@nhs.net (S.B.); anil.dhawan@kcl.ac.uk (A.D.); emer.fitzpatrick@kcl.ac.uk (E.F.); 4 Adolescent Surgical Weight Loss Clinic, Department of Child Health, King’s College Hospital, London SE5 9RS, UK; martha.ford-adams@nhs.net (M.E.F.-A.); ashishdesai@nhs.net (A.P.D.)

**Keywords:** non-alcoholic fatty liver disease, nutrition, obesity, physical activity, eating behaviour, adolescence, children

## Abstract

Non-alcoholic fatty liver disease (NAFLD) is the most common cause of chronic liver disease in children, with prevalence rising alongside childhood obesity rates. This study aimed to characterise the habitual diet and activity behaviours of children with NAFLD compared to obese children without liver disease in the United Kingdom (UK). Twenty-four biopsy-proven paediatric NAFLD cases and eight obese controls without biochemical or radiological evidence of NAFLD completed a 24-h dietary recall, a Physical Activity Questionnaire (PAQ), a Dutch Eating Behavior Questionnaire (DEBQ) and a 7-day food and activity diary (FAD), in conjunction with wearing a pedometer. Groups were well matched for age and gender. Obese children had higher BMI z-scores (*p* = 0.006) and BMI centiles (*p* = 0.002) than participants with NAFLD. After adjusting for multiple hypotheses testing and controlling for differences in BMI, no differences in macro- or micronutrient intake were observed as assessed using either 24-h recall or 7-day FAD (*p* > 0.001). Under-reporting was prevalent (NAFLD 75%, Obese Control 87%: *p* = 0.15). Restrained eating behaviours were significantly higher in the NAFLD group (*p* = 0.005), who also recorded more steps per day than the obese controls (*p* = 0.01). In conclusion, this is the first study to assess dietary and activity patterns in a UK paediatric NAFLD population. Only a minority of cases and controls were meeting current dietary and physical activity recommendations. Our findings do not support development of specific dietary/ physical activity guidelines for children with NAFLD; promoting adherence with current general paediatric recommendations for health should remain the focus of clinical management.

## 1. Introduction

Non-alcoholic fatty liver disease (NAFLD) is a condition characterised by fat accumulation in the liver with or without inflammation and fibrosis (non-alcoholic steatohepatitis; NASH), which has the potential to progress to end stage liver disease and/or hepatocellular carcinoma [1]. NAFLD is currently the most common form of chronic liver disease in children and adolescents in Western countries, with prevalence rising alongside rates of childhood obesity [2]. The gold standard for diagnosis of NASH is liver biopsy, which, due to its invasive nature and associated risks, has made establishing accurate population prevalence data for paediatric NAFLD difficult [3]. However, in an autopsy study of 742 children who died from unnatural causes in the United States (US), Schwimmer *et al.*, found a NAFLD prevalence of 9.6%, which increased to 38% in obese children [4].

Due to a lack of evidence to support pharmacological treatment options for NAFLD, diet and physical activity play a key role in NAFLD management [5,6]. First-line treatment is currently the promotion of gradual weight loss through reduced energy intake and increased physical activity, with the aim of improving liver function tests (LFTs), insulin resistance (IR), fasting glucose and lipid profiles [5,6]. However, the role of specific dietary nutrients and the influence of physical activity levels on NAFLD pathogenesis remain uncertain. Nutrients previously investigated in NAFLD pathogenesis include fructose, saturated fatty acids (SFA), mono-unsaturated fatty acids (MUFA), polyunsaturated fatty acids (PUFA) and vitamin D. Quality of dietary fat seems likely to play a role in NAFLD development with a significant body of evidence suggesting that consumption of an isocaloric diet rich in SFA may contribute to hepatic fat accumulation; whereas a diet rich in PUFA, in particular omega-3 fatty acids, may impede steatosis development [7].

More recently, there has been increasing research into the role of fructose in NAFLD pathogenesis [8]. Although high fructose intakes have been shown to alter hepatic insulin sensitivity and increase lipogenesis, whether or not this contributes to NAFLD pathogenesis independent of excess energy consumption remains uncertain [8]. Equally, vitamin D has been implicated in NAFLD development [9] with observational studies demonstrating that low vitamin D status is associated with NAFLD independently of body mass index in Australian [10], and Italian [11] paediatric cohorts. Additionally, physical activity is believed to contribute to NAFLD development with studies in both adults [12,13,14] and children [15,16] identifying high rates of physical inactivity amongst individuals with NAFLD.

In this context, the aims of this study were to characterise and compare the dietary and physical activity patterns of a group of UK children with NAFLD to obese children without evidence of liver disease; and furthermore to relate nutrient intakes to UK recommended dietary reference values and exam if excess intakes or deficiencies of nutrients previously implicated in NAFLD pathogenesis are evident in a UK paediatric cohort.

## 2. Materials and Methods

### 2.1. Participants

All participants were recruited from paediatric clinics at King’s College Hospital, London. Children with NAFLD were recruited from the Paediatric Liver Centre, whereas obese controls were recruited from a regional surgical weight loss clinic following a diagnosis of simple non-syndromic obesity and were invited into the study prior to any lifestyle intervention. All subjects were less than 18 years of age and did not consume alcohol. Cases required a diagnosis of NAFLD (initially suspected based on ultrasound or elevated liver enzymes) confirmed by liver biopsy within 3–6 months. Patients underwent a full work up for chronic liver disease including screening for Hepatitis B and C, Wilson disease (including liver copper quantification), autoimmune liver disease and alpha 1 antitrypsin deficiency. Cases were excluded in the presence of other chronic liver disease, or if they had previously received treatment for NAFLD. Controls were excluded if they had a clinical suspicion of NAFLD, based on abnormality of liver function tests or liver ultrasound. Written consent from parents/carers and assent from participants was obtained prior to entering the study. Ethical approval was granted by the National Health Service Ethics Service (10/H0808/122) and the University of Surrey Ethics Committee (EC/2010/115/FHMS).

### 2.2. Demographic and Anthropometric Data

Participant ethnicity was classified according to the Health and Social Care Information Center, Ethnic Category Code [17]. Weight (to the nearest 0.1 kg) and height without shoes (to the nearest 0.1 cm) were measured using digital scales and a commercial stadiometer (Marsden Weighing Group, Rotherham, UK). Body Mass Index (BMI) was calculated as weight (kg)/height (m^2^). BMI centile was read from the “Body mass index (BMI) 2–20 years” chart from the Royal College of Paediatrics and Child Health (RCPCH), using BMI, gender and age [18]. BMI z-score was obtained using The Children’s Hospital of Philadelphia, “Pediatric Z-Score Calculator” [19]. Weight classification was determined using BMI ranges published by the National Health Service, National Obesity Observatory [20]. Waist circumference (WC) was measured at the midway point between the top of the iliac crest and the bottom of the rib cage, using a commercial tape measure and reading to the nearest 0.5 cm [21]. If identification of these points was not possible in very overweight participants, the tape measure was placed around the abdomen at the level of the umbilicus [21]. Mid-upper arm circumference (MUAC) was recorded to the nearest 0.5 cm with a commercial tape measure and tricep skinfold thickness was measured to the nearest 0.1 mm using calipers (Holtain Tanner/Whitehouse Skinfold Caliper, Holtain Limited, Pembrokeshire, UK) [22].

### 2.3. Dietary Assessment

Dietary intakes were assessed using 24-h dietary recall and a 7-day participant-completed food diary. The 24-h dietary recall was completed at the time of consent by a research dietitian using the multiple-pass method [23]. To complete the 7-day food diary, participants were requested to document all meals, snacks and beverages consumed over 7 consecutive days; stating brand names where applicable and using household measures or food packaging information to estimate portion sizes. Participants were asked to photograph all food and beverages consumed on two of their recording days and to complete the diary during school term time. During the first 2–3 days of their recording period, participants were offered support via telephone call or home visit. After completing the food diary, participants were contacted by researchers to ascertain missing information.

Nutritional analyses were done using DietPlan6 (Forestfield Software Limited, West Sussex, UK). Dietary intakes are presented as a percentage of age- and gender-specific UK dietary reference values (DRVs) where possible [24]. Mean absolute intakes are given for nutrients that do not have DRVs. Under-reporting of dietary intake was determined using cut-offs described by Goldberg *et al.* [25]. If reported energy intake (EI) in the 7-day food diary was <1.14× Basal Metabolic rate (BMR) or 24-h recall data indicated an EI <0.92× BMR, the participant was assumed to be under-reporting. BMR was calculated using equations determined by Schofield *et al.* [26].

The 33-question Dutch Eating Behaviour Questionnaire (DEBQ) was completed by each participant at the conclusion of the 7-day recording period to assess eating styles and behaviours that may influence the likelihood of being overweight [27]. Participants responded to questions on a 5-point Likert scale. Responses are classified as expressing emotional, external or restrained eating behaviours. External eating behaviours include eating in response to food stimuli regardless of internal state of hunger or satiety. Emotional eating is consumption in response to arousal states such as anger, fear or anxiety; whereas restrained eating involves restricting food intake independently of emotional or external cues [27].

Following dietary assessment, participants received tailored advice to improve the nutritional quality of their diet, with a focus on healthy eating for weight management, via telephone call or home visit.

### 2.4. Physical Activity Assessment

Physical activity was assessed via a participant-completed 7-day physical activity diary. Participants were instructed to document all activity in 10 minute intervals on 7 consecutive days. Participants were provided with a pedometer (OMRON Step counter Walking style III, OMRON Healthcare Europe B.V., Hoofddorp, The Netherlands) and were instructed to document their daily step count on each of the 7 days. Participants received support with diary completion during the first 2–3 days of their recording period, through telephone calls and/or a home visit.

Upon return of the diaries, researchers assigned a metabolic equivalent of task (MET) to each activity using values described by Ainsworth *et al.* [28]. Activity was categorised as: sleep (0.9 METs), sedentary (1.0–1.5 METs), light (1.6–2.9 METs), moderate (3.0–5.9 METs) or vigorous (≥6 METs) [28]. Periods of time that had not being recorded were classified as unaccounted time. Absolute minutes spent in each category were calculated for each participant, along with 7-day, weekday and weekend averages for each group. Data are presented as a percentage of 24-h.

In addition, participants ≥15 years of age completed the Youth Physical Activity Questionnaire (YPAQ) and children ≤14 years completed the Children’s Physical Activity Questionnaire (C-PAQ) with the aid of a parent or guardian [29,30]. These questionnaires assessed the number of absolute minutes spent undertaking sedentary, light, moderate or vigorous exercise per week; with data presented as a percentage of total time recorded.

### 2.5. Statistical Analysis

Statistical analysis was completed using SPSS v22 (IBM SPSS Statistics, Hampshire, UK). Data are expressed as median ± interquartile range (IQR) unless otherwise specified. Due to the small sample size, non-parametric tests were used to reduce the likelihood of a type 1 error. To identify differences between groups, data were analysed using the Mann-Whitney test. Analysis of Covariance (ANCOVA) was used to correct for differences in BMI z-score between groups. Categorical baseline data and differences in the number of under-reporters were analysed using the Fisher Exact test. The Freidman test was used to assess for differences in types of eating behaviours within each group; with the Wilcoxon paired rank test used to assess differences between eating styles if *p* < 0.05. The Wilcoxon paired rank test was used to assess differences in physical activity levels and pedometer data between weekdays and weekends within each group. The Bonferroni Correction was used to determine a *p*-value to assess for group differences in macro- and micronutrient intakes. Otherwise, a *p*-value of <0.05 was considered statistically significant.

## 3. Results

### 3.1. Study Population

**Figure 1:**
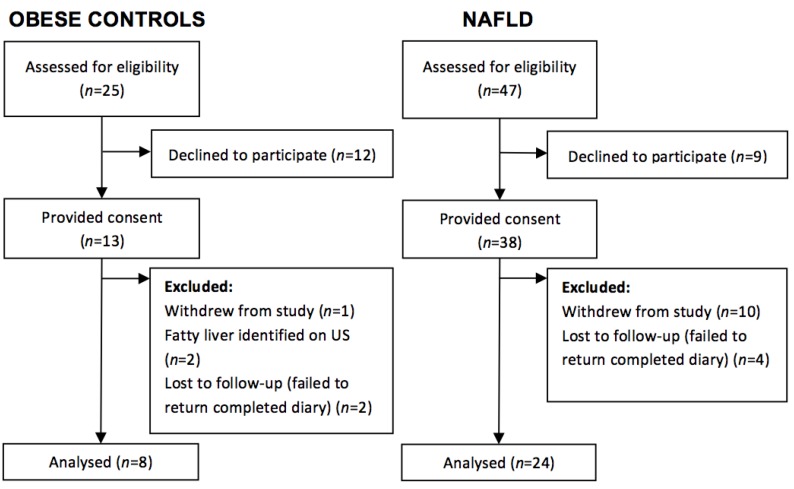
Participant flow diagram for the case-control study.

During the study period, 72 children attending King’s College Hospital paediatric clinics with NAFLD or simple obesity were approached to take part in the study. Of the 51 participants who consented to participate, 24 patients with NAFLD (63%) and 8 obese controls (61%), who had no evidence of fatty liver disease on abdominal ultrasound or liver function tests, completed the study protocol (Figure 1). Anthropometric and demographic data of the participants who completed the study protocol are outlined in Table 1. Participants ranged from 8 to 18 years of age. Groups were evenly matched for most characteristics. Although the BMI z-score (*p* < 0.01) and BMI centile (*p* < 0.01) were significantly higher in the obese control group; there were no significant differences in the distribution of weight classification between groups (*p* = 0.99). Self- (or Guardian) reported ethnicity [17] varied between groups with predominantly White British participants in the NAFLD group (79%) and Black British children in the obese group (75%).

**Table 1:** Population characteristics of NAFLD and obese control groups.

Characteristics ^1^	Unit	NAFLD (*n* = 24)	Control (*n* = 8)	*p*-Value ^2^
Gender	*n* (%)	Females 12 (50)	Females 5 (62.5)	0.69 ^a^
		Males 12 (50)	Males 3 (37.5)	
Ethnicity	*n* (%)	Black British 0 (0)	Black British 6 (75)	**<0.001 ^a^**
		White 19 (79)	White 2 (25)	
		Asian 5 (21)	Asian 0 (0)	
Weight classification	*n* (%)	Healthy Weight Range 1 (4)	Health Weight Range 0 (0)	0.99 ^a^
		Overweight 4 (17)	Overweight 1 (12.5)	
		Obese6 (25)	Obese 2 (25)	
		Extremely Obese 13 (54)	Extremely Obese 5 (62.5)	
Age	years	13.5 (*12.0*,*15.0*)	12.0 (*9.5*,*16.5*)	0.59
Weight	kg	83.3 (*71.2*,*105.0*)	86.4 (*77.2*,*136.5*)	0.27
Height	cm	163.0 (*159.1*,*170.9*)	161.6 (*148.1*,*173.7*)	0.61
BMI	kg/m^2^	31.0 (*27.1*,*36.5*)	38.4 (*30.8*,*44.1*)	0.05
BMI centile	-	99.3 (*98.0*,*99.6*)	99.6 (*98.0*,*99.6*)	**0.002**
BMI z-score	-	2.1 (*1.9*,*2.3*)	2.6 (*2.4*,*2.7*)	**0.006**
Waist circumference	cm	101.0 (*97.5*,*112.0*)	113.0 (*95.0*,*133.0*)	0.31
Triceps skinfold	mm	30.5 (*23.6*,*34.5*)	37.0 (*32.2*,*43.6*)	0.08
MUAC	cm	32.0 (*30.0*,*36.4*)	34.5 (*29.1*,*34.5*)	0.62

^1^ Characteristic is expressed as median (*1st quartile, 3rd quartile*) unless otherwise specified; ^2^ *p*-value derived from Mann-Whitney Test unless otherwise specified; ^a^ Fisher’s exact test. *p* < 0.05 in bold is considered statistically significant. BMI: Body Mass Index; MUAC: Mid Upper Arm Circumference.

### 3.2. Dietary Intakes

Overall, participants in both groups did not meet the DRV for most nutrients and had high intakes of protein, sodium, vitamin B12 and vitamin C; consuming well above the DRV (Table 2). Assessment of the 7-day diaries indicated that both the control and obese groups reported intakes of energy, fat, MUFA, PUFA, potassium, calcium, iron, zinc and folate below their DRVs. Participants in the NAFLD group consistently recorded median intakes of PUFA lower than the controls, while protein, SFA, iron, copper and zinc intakes appeared higher in NAFLD compared to controls as compared by 24-h recall. However, after correction for multiple hypotheses testing, there were no significant differences in macro- and micronutrient intakes between the NAFLD group and the obese (Table 2). There continued to be no difference between groups after correcting for BMI z-score (*p* > 0.001). Under-reporting was prevalent in both groups with 58% of participants with NAFLD and 85% of obese controls under-reporting dietary intakes, as assessed by 24-h recall. Additionally, 75% of participants with NAFLD and 87% of obese controls were deemed to have under-reported their dietary intakes using the 7-day food diary. However, the number of under-reporters did not significantly differ between groups when assessed using either the 24-h dietary recall (*p* = 0.37) or the 7-day food diary (*p* = 0.15).

**Table 2:** Nutritional intakes recorded via participant completed 7-day food diary or 24-h recall expressed as % DRV or absolute intake.

	Nutritional Intake as per 7-Day Food Diary			Nutritional Intake as per 24-h Recall		
Nutrient ^1^	NAFLD (*n* = 24)	Control (*n* = 8)	*p*-Value ^2^	NAFLD (*n* = 24)	Control (*n* = 7)	*p*-Value ^2^
**Intakes of Nutrients Expressed as % DRV**						
Energy (%)	77.4 (*67.0*,*101.2*)	86.0 (*74.4*,*114.1*)	0.41	84.7 (*56.3*,*101.7*)	52.1 (*43.6*,*93.6*)	0.10
Protein (%)	153.6 (*120.2*,*199.5*)	169.2 (*146.7*,*233.5*)	0.26	148.6 (*113.8*,*215.4*)	104.9 (*59.4*,*129.1*)	0.02
Fat (%)	82.7 (*50.1*,*105.6*)	91.7 (*67.1*,*118.4*)	0.28	77.0 (*47.3*,*105.0*)	60.7 (*39.8*,*66.5*)	0.13
SFA (%)	97.4 (*50.3*,*127.2*)	108.3 (*85.7*,*139.4*)	0.36	93.6 (*56.7*,*138.5*)	61.5 (*35.7*,*73.2*)	0.05
MUFA (%)	79.5 (*40.6*,*92.7*)	89.7 (*58.5*,*107.5*)	0.30	61.9 (*40.7*,*102.5*)	53.0 (*44.1*,*60.6*)	0.34
PUFA (%)	67.0 (*46.6*,*84.7*)	82.0 (*51.4*,*118.5*)	0.34	44.0 (*33.4*,*93.4*)	57.9 (*56.0*,*83.3*)	0.17
Carbohydrate (%)	73.0 (*56.0*,*95.2*)	87.3 (*65.4*,*95.3*)	0.51	60.2 (*44.8*,*85.2*)	51.0 (*39.3*,*83.4*)	0.44
Sodium (%)	156.3 (*110.9*,*201.4*)	167.9 (*106.2*,*199.3*)	0.86	154.1 (*91.2*,*232.0*)	109.8 (*86.1*,*131.1*)	0.16
Potassium (%)	75.9 (*50.5*,*90.8*)	75.8 (*63.2*,*97.7*)	0.32	73.3 (*51.9*,*97.23*)	52.5 (*45.3*,*78.7*)	0.08
Calcium (%)	81.5 (*51.1*,*114.6*)	79.1 (*72.5*,*106.3*)	0.79	74.0 (*32.9*,*110.8*)	57.8 (*43.7*,*77.6*)	0.48
Iron (%)	61.4 (*48.5*,*89.7*)	72.5 (*59.8*,*91.4*)	0.40	63.0 (*44.5*,*79.4*)	43.4 (*39.3*,*49.1*)	0.05
Copper (%)	100.5 (*78.2*,*148.2*)	114.1 (*99.7*,*114.1*)	0.41	89.4 (*62.5*,*153.9*)	58.8 (*52.0*,*75.0*)	0.04
Zinc (%)	78.5 (*61.0*,*118.3*)	84.4 (*78.4*,*112.9*)	0.43	62.4 (*51.4*,*103.7*)	34.3 (*33.3*,*56.6*)	0.01
Vitamin E (%)	101.0 (*63.7*,*147.5*)	120.4 (*86.0*,*186.2*)	0.43	67.5 (*40.6*,*116.1*)	109.6 (*84.2*,*155.4*)	0.30
Vitamin B12 (%)	258.3 (*167.7*,*330.5*)	250.0 (*139.3*,*545.4*)	0.76	185.0 (*115.3*,*489.2*)	127.5 (*55.3*,*190.0*)	0.09
Folate (%)	88.0 (*60.6*,*116.2*)	91.6 (*81.3*,*100.8*)	0.60	67.7 (*48.2*,*95.5*)	50.6 (*46.5*,*77.0*)	0.32
Vitamin C (%)	187.1 (*77.2*,*302.0*)	211.2 (*151.6*,*312.4*)	0.43	181.0 (*115.5*,*331.4*)	82.5 (*55.0*,*260.0*)	0.19
**Intakes of Nutrients Expressed as Absolute Intake**						
Starch (g)	119.4 (*84.7*,*158.2*)	127.4 (*101.7*,*137.6*)	0.83	105.4 (*80.3*,*136.9*)	107.2 (*52.3*,*148.2*)	0.67
Sugar (g)	87.5 (*49.5*,*107.1*)	93.0 (*82.2*,*102.9*)	0.49	63.5 (*44.9*,*125.0*)	49.9 (*22.8*,*80.1*)	0.24
NMES (g)	15.6 (*5.1*,*25.0*)	20.8 (*11.4*,*28.7*)	0.46	16.0 (*2.0*,*36.6*)	9.8 (*0.0*,*21.4*)	0.32
Fructose (g)	12.7 (*8.9*,*18.4*)	16.7 (*10.9*,*19.4*)	0.62	12.5 (*6.3*,*20.2*)	9.1 (*2.1*,*19.4*)	0.38
NSP (g)	10.7 (*8.2*,*13.1*)	10.1 (*6.3*,*12.3*)	0.41	8.7 (*7.0*,*11.3*)	4.1 (*4.0*,*10.9*)	0.14
Carotene (g)	1.9 (*0.7*,*3.1*)	3.0 (*0.6*,*5.8*)	0.32	1.0 (*0.2*,*2.3*)	0.4 (*0.04*,*1.3*)	0.22
Vitamin D (µg)	1.7 (*1.3*,*2.2*)	3.5 (*1.0*,*4.4*)	0.32	1.8 (*1.2*,*3.6*)	1.9 (*0.3*,*2.9*)	0.45
Omega-3 Fatty Acids (g)	0.5 (*0.3*,*0.9*)	0.5 (*0.15*,*1.4*)	0.91	0.5 (*0.2*,*0.8*)	0.3 (*0.2*,*0.4*)	0.59

^1^ Data are expressed as median (*1st quartile, 3rd quartile*); ^2^ *p*-values from Mann-Whitney Test with Bonferroni Correction; *p* < 0.001 is considered statistically significant. DRV: Dietary Reference Value; MUFA: Monounsaturated Fatty Acids; NSP: non-starch polysaccharide, NMES: Non milk extrinsic sugars; PUFA: Polyunsaturated Fatty Acids; SFA: Saturated Fatty Acids.

Although, there were no significant differences in the percent of energy derived from total fat, SFA, MUFA or PUFA between groups, both the obese control and NAFLD group exceeded the recommended percent of energy derived from saturated fat and received an insufficient proportion of energy from PUFA and MUFA (Table 3). However, both groups met the recommendations for total fat intake deriving less than 35% of energy from fat.

**Table 3:** Percentage of energy derived from total fat, SFA, MUFA and PUFA recorded via participant completed 7-day food diary.

Nutrients Contributing to Energy Intake ^1^	Recommended Daily Allowance (Expressed as Percentage (%) Total Energy Intake)	NAFLD (*n* = 24)	Control (*n* = 8)	*p*-Value ^2^
Total Fat	<35	33.1 (*28.2*,*38.6*)	34.1 (*32.6*,*36.6*)	0.69
SFA	<11	11.7 (*9.6*,*13.7*)	12.4 (*11.4*,*13.2*)	0.31
MUFA	13	10.4 (*8.8*,*13.6*)	11.9 (*8.7*,*12.7*)	0.68
PUFA	6.5	4.9 (*4.2*,*6.2*)	4.9 (*4.2*,*6.8*)	0.88

^1^ Data are expressed as median (*1st quartile, 3rd quartile*); ^2^ *p*-values from Mann-Whitney Test. *p* < 0.05 is considered statistically significant.

**Figure 2:**
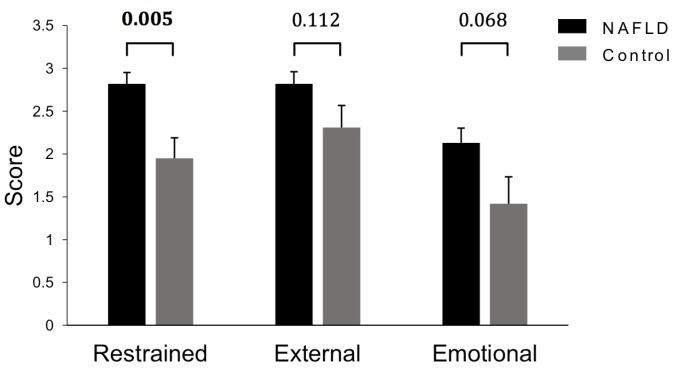
Eating styles in NAFLD and obese children as assessed by Dutch Eating Behaviour Questionnaires. Data adjusted for differences in BMI Z-score using ANCOVA and are expressed as estimated marginal mean ± standard error of the mean (SEM). NAFLD (*n* = 23) Control (*n* = 8).

After controlling for differences in BMI z-score, responses to the Dutch Eating Behaviour Questionnaire indicated that restrained eating behaviours were significantly higher in the NAFLD group (*p* = 0.005) (Figure 2). In addition, NAFLD patients reported comparatively higher scores for external (*p* = 0.112) and emotional eating behaviours (*p* = 0.068).

### 3.3. Physical Activity Levels

**Figure 3:**
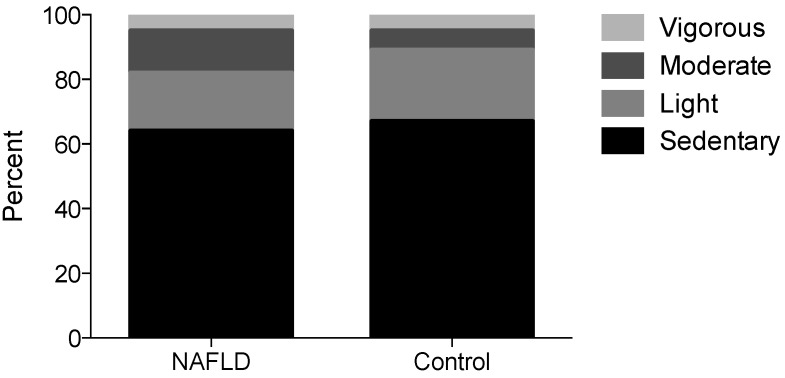
Physical activity levels expressed as amount of time per week (%) calculated using the Youth or Children’s Physical Activity Questionnaire. Data are expressed as percent time per week. NAFLD (*n* = 16) Control (*n* = 8).

Physical activity levels, assessed using both the 7-day diary and PAQ, indicated that participants spent a large proportion of their time in sedentary behaviour with minimal vigorous activity (Table 4 and Figure 3). The 7-day activity diary suggested that NAFLD patients spent more time on weekdays in moderate activity (*p* = 0.06) while controls had more weekday time (22% *vs.* 5%) unaccounted for (Table 4). Interestingly, twenty-five percent of participants in the NAFLD group reported meeting the UK physical activity requirements of 60 min of moderate to vigorous activity per day [31], using the physical activity levels recorded in the 7-day activity diary. In contrast, none of the obese controls met these guidelines. However, there were no significant differences between groups in the median amount of time per day (Table 4) or per week (Figure 3) spent undertaking sedentary, light, moderate or vigorous activity after controlling for BMI z-score.

**Table 4:** Physical Activity Levels expressed as amount of time (%) per day recorded using 7-day activity diary.

Physical Activity Levels ^1^ (% per Day)		NAFLD (*n* = 22–24)	Control (*n* = 8)	*p*-Value ^2^
Sedentary (MET 1.0–1.5)	7-day Average	25 (*16,32*)	20 (*13,28*)	0.43
	Weekday Average	22 (*16,30*)	23 (*16,31*)	0.95
	Weekend Average	29 (*6,36*)	16 (*10,22*)	0.22
Light (MET 1.6–2.9)	7-day Average	18 (*9,23*)	13 (*5,26*)	0.73
	Weekday Average	25 (*11,28*)	16 (*6,31*)	0.41
	Weekend Average	6 (*1,14*)	8 (*3,13*)	0.71
Moderate (MET 3.0–5.9)	7-day Average	5 (*3,7*)	3 (*3,4*)	0.31
	Weekday Average	5 (*3,7*)	2 (*1,4*)	0.06
	Weekend Average	4 (*1,8*)	7 (*5,11*)	0.13
Vigorous (MET > 6)	7-day Average	2 (*0,3*)	1 (*0,2*)	0.28
	Weekday Average	2 (*0,4*)	1 (*0,2*)	0.19
	Weekend Average	0 (*0,3*)	0 (*0,1*)	0.48
Sleep (MET 0.9)	7-day Average	40 (*38,43*)	42 (*38,46*)	0.33
	Weekday Average	39 (*36,42*)	41 (*37,41*)	0.32
	Weekend Average	44 (*42,46*)	45 (*39,45*)	0.62
Unaccounted	7-day Average	7 (*7,22*)	19 (*5,32*)	0.15
	Weekday Average	5 (*5,10*)	22 (*5,30*)	0.08
	Weekend Average	6 (*0,21*)	20 (*9,28*)	0.16

^1^ Data are expressed as median (*1st quartile, 3rd quartile*); ^2^ *p*-values from Mann-Whitney Test. *p* < 0.05 is considered statistically significant. MET: Metabolic Equivalent of Task.

**Figure 4:**
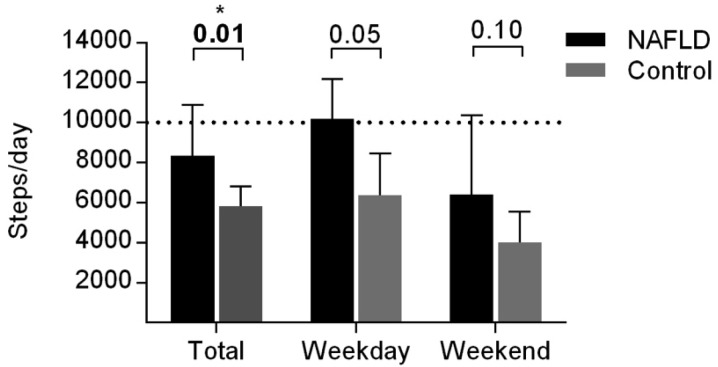
Average steps recorded using pedometer during participant completed 7-day physical activity diary. Data are unadjusted and expressed as median + 3rd quartile. *p*-values from Mann-Whitney Test. *p* < 0.05 is considered statistically significant. Dashed line represents 10,000 steps; minimum number of steps required by adolescence to meet 60 min/day moderate-to-vigorous physical activity recommendation [32].

In addition to the physical activity diary, participants recorded step counts assessed by a pedometer. On average, the NAFLD group took more steps than the obese controls (*p* = 0.01) (Figure 4). There was no significant difference between the number of steps taken on a weekday and a weekend days in the NAFLD group (*p* = 0.062), whereas participants in the obese control group took more steps on weekday compared to a weekend (*p* = 0.046).

## 4. Discussion

This is the first study to compare macro- and micronutrient intakes, eating behaviours and physical activity behaviours of UK paediatric NAFLD patients to obese children without evidence of liver disease. Anthropometric measurements were similar between groups with the exception of BMI z-score and BMI centile, which were higher in the obese control group. Accumulation of visceral fat has been implicated in the development and progression of NAFLD in both adults and children, suggesting that distribution of adipose tissue may be more influential in determining NAFLD risk than overall adiposity [33,34]. Despite no difference in waist circumference, further exploration and comparison of body composition between groups may have provided insight into whether visceral fat mass influenced NAFLD development in this cohort. A significant association between group and self/guardian-reported ethnicity was identified. Ethnic variation in NAFLD prevalence has previously been established, with studies in the USA noting higher prevalence of NAFLD amongst Hispanics, followed by white European-Americans, with lowest disease prevalence observed in black African-Americans [35]. This was subsequently associated with ethnic variation in the prevalence of allelic variants of the patatin-like phospholipase 3 (PNPLA3) gene; with the PNPLA3 rs738409 (G) allele strongly associated with increased hepatic steatosis and inflammation [36]. Given that the majority of participants with NAFLD were White British, compared to a majority of Black British children in the control group, it would be interesting to explore whether a higher prevalence of PNPLA3 rs738409 (G) exists in this NAFLD cohort.

After controlling for differences in BMI and correcting for multiple hypotheses testing, we found no significant differences in macro- or micronutrient intake between groups. This includes nutrients such as fructose, fatty acids and vitamin D previously implicated in NAFLD pathogenesis. The fact that there were no statistical differences in fructose intake between groups opposes the results of a number of previous studies noting increased fructose consumption in NAFLD patients [15,37,38,39]. However, our findings are consistent with a 2014 systematic review and meta-analysis where reviewers concluded that any observed detrimental effects of excess dietary fructose might be attributable to excessive energy intake rather than increased fructose consumption in isolation [40]. While there were no differences between the NAFLD and obese groups, reported intakes of MUFA and PUFA were below UK recommendations; with low intakes of omega-3 fatty acids. Considering that previous studies have linked MUFA with reduced hepatic steatosis [41] and improved insulin sensitivity [41], and PUFA (in particular omega-3) with improvements in LFTs [42,43], serum triglyceride (TG) and fasting blood glucose levels [42]; dietary deficiencies in MUFA and PUFA are of concern. Education on accessible dietary sources of unsaturated fatty acids should remain a priority in this population. Equally, although there were no statistical differences in vitamin D consumption between groups, vitamin D intakes (1.7–3.5 µg/day) were well below the new proposed UK recommended nutrient intake for vitamin D of 10 µg/day [44]. As vitamin D is derived both exogenously, from dietary sources, and endogenously, by dermal synthesis following exposure to UVB radiation [9], further research should be completed to assess the influence of both vitamin D intake and serum 25 (OH)D levels on NAFLD pathogenesis in a paediatric UK cohort.

A key observation was the severe degree of under-reporting dietary intakes. Under-reporting is known to be particularly common in obese subjects, characterised by reporting relatively low intakes of foods which may be perceived as socially undesirable [45,46]. In addition, under-reporting may have been unintentional, with reliance on memory and inexperience of portion size estimation known to limit accurate dietary recall in children [47]. Despite a lack of significant difference in macro- and micronutrient intake between groups, the majority of children in this cohort were consuming well below the DRV for most nutrients. Although, rates of under-reporting were high and reported intake may be lower than actual intake, there should be a focus on promoting intake of a wide range of nutritious foods to assist children in consuming sufficient macro- and micronutrients to meet UK recommendations. While certain eating behaviours are closely linked with obesity [48], few studies have assessed eating behaviours amongst children with NAFLD. Interestingly, in this cohort restrained eating behaviours were higher in the NAFLD group after controlling for BMI, which suggests that children with NAFLD are more cautious of their dietary intake. Hattar *et al.* [16] compared nutrition attitudes of obese children with NASH to obese control children and found 35% of the NASH group consistently read nutritional information labels on food/beverages, compared to 0% of controls. Consequently, the greater awareness of dietary intake by children in the present study may be a consequence of the NAFLD diagnosis.

Equally notable, children with NAFLD in this study were more physically active and took more steps than their obese counterparts. Indeed, twenty-five percent of participants with NAFLD met the UK physical activity guidelines [31], whereas all participants in the control group failed to meet this recommendation. While the most appropriate duration and intensity of physical activity for the treatment of NAFLD remains to be established, a number of adult studies have noted metabolic benefits of regular, moderate-intensity activity in NAFLD patients, including decreases in visceral adiposity and hepatic TG concentration [49]. As our study noted no clear differences in activity levels between participants with NAFLD and obese controls, general paediatric guidelines for physical activity should be encouraged in all overweight/obese children. Additionally, there should be a particular emphasis on encouraging physical activity outside of school hours, as we noted a substantial decline in steps taken on weekends compared to weekdays.

A key strength of this study was the use of liver biopsy to diagnose NAFLD rather than reliance on LFTs or ultrasound, considered to be low sensitivity diagnostic tools [5]. Although controls were not precisely BMI-matched to NAFLD participants, both groups were of similar weight status. This is of key importance in identifying risk factors other than obesity, which may contribute to NAFLD development. An additional strength was the high levels of support received by participants in completion of food and activity diaries, through home visits and telephone calls, maximising accuracy of diary completion. On the other hand, a limitation of this study was the low number of controls compared to NAFLD participants increasing the risk of a Type 2 error. It was much more difficult to successfully recruit control subjects, reflecting a wider issue of low compliance in overweight/obese children with weight reduction initiatives [50]. Additionally, due to the specialised nature of the paediatric NAFLD clinic, referrals were received from across south-west England whereas the patients for the multidisciplinary obesity clinic has a significant proportion of individuals from a region in London with a large population of individuals from African and Caribbean descent [51], skewing the ethnic distribution between groups. Lastly, some of the subjects might have previously seen a dietitian, or received dietary advice upon diagnosis, leading to altered eating and physical activity behaviors during completion of the study protocol. However, all patients were assessed according to the growth trajectory on Royal College of Paediatrics and Child Health growth charts to ensure no loss of growth centiles, indicating weight loss, had been seen in the previous months during diagnosis.

## 5. Conclusions

In summary, no differences in macro- or micronutrient intakes were observed between children with NAFLD and obese controls in this study. Under-reporting in both groups was prevalent. Restrained eating behaviours were significantly higher in the NAFLD group, who were also more physically active and took more steps than their obese counterparts. Further research is required to confirm the role of particular nutrients in the development and progression of NAFLD and to establish optimal physical activity guidelines for children with NAFLD. Only a minority of cases and controls were meeting current dietary and physical activity recommendations and general healthy eating advice, alongside a minimum of 60 min moderate-vigorous physical activity per day should continue to be encouraged [31]. Additionally, the fact that children in the control group were of significantly higher BMI than children in the NAFLD group suggests excess body weight is not the only driving factor in NAFLD development. Genetic susceptibility, ethnicity, the gut microbiome and visceral fat accumulation may all play a role in the risk of developing NAFLD.
